# The Impact of Colostomy on Inpatient Outcomes Following Primary Total Knee Arthroplasty

**DOI:** 10.7759/cureus.65900

**Published:** 2024-07-31

**Authors:** Lemuelson Aryeetey, Andrew J Hinkle, Sergio Huerta, Senthil Sambandam

**Affiliations:** 1 Department of Orthopaedic Surgery, University of Texas Southwestern Medical Center, Dallas, USA

**Keywords:** acute kidney injury, infection, total knee arthroplasty, inpatient complications, colostomy

## Abstract

Introduction

The inpatient postoperative outcomes of patients with colostomies following primary total knee arthroplasty (TKA) have not been well studied in the literature. The purpose of this study was to analyze how colostomy impacts the immediate postoperative outcomes of TKA. Our null hypothesis is that after correcting for common variables, a colostomy does not predispose patients undergoing TKA to surgical site infections (SSIs) and periprosthetic infections.

Methods

The National Inpatient Sample database was used to retrieve information on colostomy patients and patients without a colostomy who had undergone primary TKA from 2016 to 2019. Patients with colostomies were matched to a cohort of non-colostomy control groups in a 1:1 propensity score algorithm by age, sex, race, and pertinent comorbidities. Patient demographic characteristics, comorbidities, length of hospital stay (LOS), total hospital charges, and inpatient complications were compared.

Results

Following propensity score matching, 399 patients with colostomies were compared to 385 patients without a colostomy (control). The colostomy group had a prolonged LOS (3.15 ± 2.67 vs 2.44 ± 3.15 days, p<0.001) compared to the control group. Also, the colostomy group had significantly higher incidences of acute kidney injury (AKI) (6.02% vs 1.56%, odds ratio (OR): 4.04, 95% confidence interval (CI): 1.63-10.00, p<0.001), blood loss anemia (20.55% vs 13.25%, OR: 1.69, 95% CI: 1.16-2.48, p=0.008), and blood transfusions (4.01% vs 0.26%, OR: 16.04, 95% CI: 2.12-121.56, p<0.001). There was no difference in periprosthetic infection, superficial SSI, or deep SSI.

Conclusion

Patients with colostomies face a notably higher risk of experiencing AKI, blood loss anemia, and blood transfusion requirements during the immediate postoperative period following primary TKA. Despite the perceived risk of postoperative infection in colostomy patients, this patient population is not at an increased risk of developing periprosthetic infection, superficial SSI, or deep SSI following TKA.

## Introduction

The prevalence of ostomy, a surgical anastomosis of a part of the gastrointestinal tract and the anterior abdominal wall, is estimated to be 750,000 per one million, with approximately 100,000 people undergoing this operation each year [1,2]. Colostomy, a type of ostomy involving the diversion of the colon through an opening in the abdominal wall, is performed to manage a variety of complications associated with multiple conditions including inflammatory bowel disease, colorectal cancer, congenital anomalies, diverticulitis, and intestinal trauma [3,4]. Electively, colostomies might also be performed for patients with chronic constipation, incontinence, or patients with spinal cord injuries with decubitus ulcers [5-7]. Significantly, a colostomy has been demonstrated to extend the life expectancy of patients in a number of these cohorts [8,9]. As life expectancy increases among colostomy patients, an associated rise in elective surgical procedures including total knee arthroplasty (TKA) is anticipated. The average age of patients with colostomies is 70 years while the average age of a patient with a primary TKA recipient is 68 years [10,11]. The convergence of these ages would suggest the increased likelihood of a growing demand for TKA within the population of patients with colostomies. Understanding the implications of colostomy following TKA is crucial for optimizing patient outcomes in this patient population.

Furthermore, periprosthetic joint infection following TKA can present as a devastating complication, leading to severe health implications, with a five-year mortality rate exceeding that of female breast cancer and prostate cancer [12]. The presence of an ostomy introduces the potential perception of a risk of fecal contamination of the surgical field, which might lead to perioperative infectious complications [13]. The incidence of surgical site infection (SSI) in colostomy patients who undergo elective surgery varies from 3.8% to 27.2% [14-16]. However, the incidence of SSI in colostomy patients who undergo primary TKA is not currently reported in the literature.

The only study conducted to analyze the effects of colostomy on total joint arthroplasty outcomes was performed by Yang and Sambandam, in which they reported that patients with colostomies who undergo total hip arthroplasty (THA) are at significant risk of periprosthetic infection and dislocation, longer hospital stay, and greater hospital cost than patients without a colostomy [17]. The purpose of this study is to characterize the immediate postoperative outcomes in colostomy patients undergoing TKA. We hypothesize that having a colostomy will result in prolonged length of stay (LOS) and greater incidence of postoperative medical complications and surgical complications, specifically, periprosthetic infection, superficial SSI, and deep SSI following TKA.

## Materials and methods

The National Inpatient Sample (NIS) database, a component of the Healthcare Cost and Utilization Project sponsored by the Agency for Healthcare Research and Quality, stands as the United States (U.S.) most extensive publicly available all-payer database for inpatient care. Annually, the NIS aggregates data from approximately seven million hospital admissions spanning nearly 1000 hospitals in 48 states and the District of Columbia. The NIS represents an approximately 20% stratified sample of all discharges from U.S. hospital admissions. Its large sample size allows for the analyses of rare conditions, specific patient populations, and uncommon treatments [18].

This study utilized the NIS database to retrieve information on patients who had undergone primary TKA. Patients with the International Classification of Diseases, Tenth Revision, Clinical Modification (ICD-10-CM) codes for partial knee arthroplasty, and revision of TKA were excluded from this study. The total number of patients identified was stratified based on the presence or absence of a colostomy. Patient demographic characteristics, such as age, sex, and race, as well as comorbidities, such as obesity, complicated diabetes, uncomplicated diabetes, and tobacco use, were compared. The reporting of race, as self-identified by patients, was included as data to highlight the inequities experienced by different racial groups. Furthermore, LOS, total hospital charges in U.S. dollars, and inpatient medical and surgical complications were also compared. Complications of interest included inpatient mortality, pneumonia, acute kidney injury (AKI), deep vein thrombosis, pulmonary embolism, periprosthetic dislocation, periprosthetic infection, periprosthetic mechanical dislocation, periprosthetic fracture, superficial and deep SSI, wound dehiscence, myocardial infarction, blood loss anemia, and blood transfusions. These data elements were derived using the ICD-10-CM/Procedure Coding System (PCS) (Table 1). The NIS dataset is deidentified, fulfilling the criteria for exemption from International Review Board approval for this study.

**Table 1:** ICD-10 Codes Used for Data Retrieval ICD-10: International Classification of Diseases, Tenth Revision

Colostomy	Comorbidities	Medical Complications	Surgical Complications
Z93	Obesity E660, E6601, E6609, E661, E662, E668, E669, Z6830, Z6831, Z6832, Z6833, Z6834, Z6835, Z6836, Z6837, Z6838, Z6839 Diabetes without complications E119 Diabetes with complications E1169 Tobacco-related disorder Z87891	Acute renal failure N170, N171, N172, N178, N179 Myocardial infarction I2101, I2102, I2111, I2113, I12114, I12119, I2121, I12129, I21A1 Blood loss anemia D62 Pneumonia J189, J159, J22 Blood transfusion 30233N1 DVT I82401,I82402, I82403, I82409, I82411, I82412, I82413, I82419, I82421, I82422, I82423, I82429, I82431, I82432, I82433, I82439, I82441, I82442, I82443, I82449, I82491, I82492, I82493, I82499, I824Y1, I824Y2, I824Y3, I824Y9, I824Z1, I824Z2, I824Z3, I824Z4 Pulmonary embolism I2602, I2609, I2692, I2699	Periprosthetic fracture T84012A, T84013A, T84018A, T84019A, T84099A, T84029A, M96661, M96662, M96669, M96671, M96672, M96679, M9669, M9711XA, M9712XA Periprosthetic dislocation T84022A, T84023A, T84028A, T84029A Periprosthetic mechanical complication T84092A, T84093A, T84098A, T84099A Periprosthetic infection T8450XA, T8453XA, T8454XA, T8459XA Superficial SSI T8141XA Deep SSI T8142XA Wound Dehiscence T8130XA, T8131XA, T8132XA

Statistical analysis

Chi-squared test was applied for categorical variables and Student’s t-tests were applied for continuous variables. A 1:1 propensity matching was conducted using pre-operative parameters, such as age, sex, race, and pertinent comorbidities. The odds ratio (OR) and their associated 95% confidence intervals (CI) for the inpatient complications were calculated as a ratio of the incidence in the control group to the incidence in the colostomy group. Fisher’s exact test was used for incidence values less than five. A two-tailed p-value ≤ 0.05 was deemed statistically significant. All statistical analysis was performed using Statistical Package for the Social Sciences (IBM SPSS Statistics for Windows, IBM Corp., Version 27.0, Armonk, NY).

## Results

Demographic characteristics and comorbidities

A total of 558,371 patients with pertinent preoperative characteristics who underwent primary TKA between 2016 and 2019 were compiled using the NIS database. Among this group of patients, 399 had colostomy and 557,972 formed the non-colostomy control group. The mean age of patients with colostomies was 70.92 ± 9.40 while the mean age of the control group was 66.71 ± 9.51, p<0.001. There was no statistically significant difference in terms of distribution of sex, obesity, race, uncomplicated and complicated diabetes, and tobacco use. The 1:1 propensity score matching resulted in 399 patients in the colostomy group and 385 patients in the non-colostomy control group. There was no significant difference in demographic characteristics and comorbidities between the two groups (Table 2).

**Table 2:** Patient Demographic Characteristics and Comorbidities (Unmatched and Matched Cohorts) SD: standard deviation; NA: not applicable The values were expressed as n (%). * Obesity is defined as a body mass index ≥ 30. Bold values indicate statistical significance (P<0.05).

Characteristics	Unmatched Colostomy (n=399)	Unmatched Control (n=557972)	P-value	Matched Colostomy (n=399)	Matched Control (n=385)	P-value
Mean age at admission, years (SD)	70.92 (9.40)	66.71 (9.51)	<0.001	70.92 (9.40)	70.45 (9.00)	0.482
Sex (women)	230 (57.64%)	343190 (61.51%)	0.122	230 (57.64%)	222 (57.66%)	1.000
Obesity*	129 (32.33%)	172532 (30.94%)	0.553	129 (32.33%)	121 (31.42%)	0.818
Race						
White	335 (83.96%)	4348822 (81.15%)	0.097	335 (87.01%)	335 (87.01%)	1.000
Black	22 (5.51%)	44817 (8.36%)	-	22 (5.51%)	22 (5.51%)	-
Hispanic	18 (4.51%)	33576 (6.27%)	-	18 (4.51%)	18 (4.51%)	-
Asian or Pacific Islander	5 (1.25%)	8246 (1.54%)	-	5 (1.30%)	5 (1.30%)	-
Native American	1 (0.26%)	2530 (0.47%)	-	1 (0.26%)	1 (0.26%)	-
Other	4 (1.00%)	11878 (2.22%)	-	4 (1.04%)	4 (1.04%)	-
Diabetes without complications	55 (13.78%)	82409 (14.77%)	0.617	55 (13.78%)	54 (14.03%)	1.000
Diabetes with complications	0	1404 (0.25%)	0.632	0	0	NA
Tobacco use	68 (17.04%)	88301 (15.83%)	0.496	68 (17.04%)	65 (16.88%)	1.000

LOS and hospital charges

The colostomy group had prolonged LOS, averaging 3.15 days while the control group averaged 2.35 days, p<0.001. The colostomy group incurred $3,862.81 more in total hospital charges than the control group, but this did not reach statistical significance ($68670.94 ± $41762.72 vs $64808.13 ± $45837.38, p=0.092). After propensity score matching, the colostomy group had a statistically significant prolonged LOS (3.15 vs 2.44, p<0.001), but there was no statistically significant difference in total hospital charges (Table 3).

**Table 3:** Comparison of Length of Stay and Hospital Charges of the Unmatched and Matched Cohorts The values were expressed as mean (SD). Bold values indicate statistical significance (P<0.05).

Characteristics	Unmatched Colostomy (n=399)	Unmatched Control (n=557972)	P-value	Matched Colostomy (n=399)	Matched Control (n=385)	P-value
Length of stay, days	3.15 (2.67)	2.35 (1.93)	<0.001	3.15 (2.67)	2.44 (3.15)	<0.001
Total Charges in U.S. Dollars	68670.94 (41762.72)	64808.13 (45837.38)	0.092	68670.94 (417627.22)	62959.34 (45746.44)	1.825

Inpatient postoperative complications and in-hospital mortality

The incidence of AKI was greater in the colostomy group, 6.02%, than in the control group, 1.98% (OR: 3.16, 95% CI: 2.09-4.78, p<0.001). There was no difference in periprosthetic infection, superficial SSI, or deep SSI. The colostomy group also had a higher incidence of blood loss anemia (20.56% vs 15.32%, OR: 1.43, 95% CI: 1.12-1.83, p=0.004) and blood transfusions (4.01% vs 1.48%, OR: 2.79, 95% CI: 1.69-4.60, p<0.001) than the control group. There was no statistically significant difference among the two groups on other postoperative complications (Table 4). After propensity score matching, the colostomy group had a greater incidence of AKI (6.02% vs 1.56%, OR: 4.04, 95% CI: 1.63-10.00, p<0.001), blood loss anemia (20.55% vs 13.25%, OR: 1.69, 95% CI: 1.16-2.48, p=0.008), and blood transfusions (4.01% vs 0.26%, OR: 16.04, 95% CI: 2.12-121.56, p<0.001). There was no difference in periprosthetic infection, superficial SSI, or deep SSI (Table 5).

**Table 4:** Comparison of Inpatient Postoperative Complications and In-Hospital Mortality (Unmatched Cohorts) DVT: deep vein thrombosis; SSI: surgical site infection The values were expressed as n (%). * Odds ratios, 95% confidence interval, and significant values could not be calculated due to an incidence of 0 in one or both groups. Bold values indicate statistical significance (P<0.05).

Complications	Unmatched Colostomy (n=399)	Unmatched Control (n=557972)	Odds Ratio (95% Confidence Interval)	P-value
Hospital Mortality	1 (0.25%)	197 (0.04%)	7.11 (1.00, 50.85)	0.132
Acute Kidney Injury	24 (6.02%)	11061 (1.98%)	3.16 (2.09, 4.78)	<0.001
Myocardial Infarction	0	109 (0.02%)	*	*
Blood Loss Anemia	82 (20.56%)	85475 (15.32%)	1.43 (1.12, 1.83)	0.004
Pneumonia	2 (0.50%)	1086 (0.19%)	2.58 (0.64, 10.38)	0.183
DVT	1 (0.25%)	1261 (0.23%)	1.11 (0.16, 7.90)	0.595
Pulmonary Embolism	2 (0.50%)	1236 (0.22%)	2.27 (0.57, 9.12)	0.222
Periprosthetic Fracture	1 (0.25%)	2362 (0.42%)	0.59 (0.08, 4.21)	1.00
Periprosthetic Dislocation	4 (1.00%)	4258 (0.76%)	1.32 (0.49, 3.53)	0.554
Periprosthetic Mechanical Complication	1 (0.25%)	4503 (0.81%)	0.31 (0.04, 2.20)	0.390
Periprosthetic Infection	8 (2.00%)	5794 (1.04%)	1.95 (0.97, 3.93)	0.075
Superficial SSI	0	26 (0%)	*	*
Deep SSI	0	8 (0%)	*	*
Wound Dehiscence	0	530 (0.09%)	*	*
Blood Transfusion	16 (4.01%)	8238 (1.48%)	2.79 (1.69, 4.60)	<0.001

**Table 5:** Comparison of Inpatient Postoperative Complications and In-Hospital Mortality (Matched Cohorts) DVT: deep vein thrombosis; SSI: surgical site infection The values were expressed as n (%). * Odds ratios, 95% confidence interval, and significant values could not be calculated due to an incidence of 0 in one or both groups. Bold values indicate statistical significance (P<0.05).

Complications	Matched Colostomy (n=399)	Matched Control (n=385)	Odds Ratio (95% Confidence Interval)	P-value
Hospital Mortality	1 (0.25%)	0	*	*
Acute Kidney Injury	24 (6.02%)	6 (1.56%)	4.04 (1.63, 10.00)	0.001
Myocardial Infarction	0	0	*	*
Blood Loss Anemia	82 (20.55%)	51 (13.25%)	1.69 (1.16, 2.48)	0.008
Pneumonia	2 (0.50%)	4 (1.04%)	0.48 (0.09, 2.64)	0.444
DVT	1 (0.25%)	1 (0.26%)	0.97 (0.06, 15.48)	1.000
Pulmonary Embolism	2 (0.50%)	2 (0.52%)	0.97 (0.14, 6.89)	1.000
Periprosthetic Fracture	1 (0.25%)	2 (0.52%)	0.48 (0.04, 5.33)	0.618
Periprosthetic Dislocation	4 (1.00%)	2 (0.52%)	1.94 (0.35, 10.65)	0.687
Periprosthetic Mechanical Complication	1 (0.25%)	3 (0.78%)	0.32 (0.03, 3.09)	0.365
Periprosthetic Infection	8 (2.01%)	5 (1.30%)	1.56 (0.50, 4.80)	0.579
Superficial SSI	0	0	*	*
Deep SSI	0	0	*	*
Wound Dehiscence	0	1 (0.26%)	*	*
Blood Transfusion	16 (4.01%)	1 (0.26%)	16.04 (2.12, 121.56)	<0.001

## Discussion

The current literature lacks studies that address the outcomes of colostomy patients after primary TKA. To address this paucity, we obtained data from the NIS database and conducted both unmatched and matched analyses to compare inpatient postoperative outcomes after TKA between patients with and without a colostomy. The findings from this study demonstrate that colostomy patients who undergo TKA have an increased risk of AKI, blood loss anemia, and blood transfusion requirement even after propensity score matching. Additionally, colostomy patients experienced prolonged LOS. The incidence of periprosthetic infection, superficial SSI, and deep SSI were similar between the colostomy group and the non-colostomy control group.

Our analysis shows that colostomy patients have four-fold increased odds of AKI, two-fold increased odds of blood loss anemia, and 16-fold increased odds of blood transfusion requirement. Limited data has been published concerning the incidence of these complications among colostomy patients after undergoing elective surgery, especially with regard to orthopedic surgery. However, the findings in this study were in disagreement with the findings by Yang and Sambandam in their analysis of complications in colostomy patients after THA. They found no significant difference in blood loss anemia, blood transfusion requirement, and AKI in colostomy patients undergoing THA [17]. This study utilized the same database and methodology thus controlling for sampling and chronological bias when comparing the two studies.

The mean LOS in this study was 3.15 days for the colostomy group compared to 2.44 days for the control group. Although there may be a statistical link between colostomy and extended hospital stays following TKA, the actual effect on patient outcomes may be insignificant or insufficient to necessitate considerable clinical concern. This difference is likely a reflection of underlying co-morbidities in colostomy patients compared to the general TKA patient population.

The etiology of AKI in colostomy patients may be due to several reasons with considering both acute and chronic processes in a patient’s underlying disease. In general, colostomy patients are at risk for decreased fluid and electrolyte absorption due to diversion from the remainder of the large intestine [19]. A decreased capacity to absorb water in the large intestine in the perioperative period may lead to decreased renal perfusion because of dehydration. Additionally, these patients are at heightened risk for fluid loss via the stoma which may contribute to the overall risk of AKI [20]. Patients and clinicians should monitor for signs and symptoms of blood and sensitive fluid loss, such as oliguria, nausea, increased thirst, dry mouth, concentrated urine, and lightheadedness [21].

Similarly, the perioperative risk of anemia in colostomy patients after elective surgery has not been well studied. The increased risk of anemia and subsequent transfusion requirement is likely a reflection of the patient’s underlying disease. Colostomies are created for several underlying conditions including colorectal cancer and inflammatory bowel disease that independently cause blood loss, vitamin deficiencies, or malnutrition [19]. Specifically, the inflammatory bowel disease population is at risk for folate and vitamin B12 deficiency which may contribute to anemia [19]. While it is unlikely that patients would have acute stomal bleeding as a result TKA, this should be monitored postoperatively and care should be taken to protect the ostomy site during patient transfers and positioning.

Contrary to our hypothesis, there was no statistically significant difference between the colostomy patients and the control group concerning inpatient surgical complications, such as periprosthetic infection, superficial SSI, and deep SSI. This is in contradiction to the findings of Yang and Sambandam, who reported an increased risk of periprosthetic infection among colostomy patients undergoing THA [17]. However, they did not comment on the incidence of SSIs. Other studies analyzing surgical outcomes in patients with colostomies who undergo surgical procedures agree with the findings of this study. In a retrospective study comparing outcomes of patients with colostomies undergoing either emergent or nonemergent parastomal hernia repair, Gregg et al. reported no significant difference in superficial SSI and deep SSI [15]. Likewise, in a retrospective study analyzing complications following parastomal hernia repair, Khan et al. found no significant difference in wound complications, such as superficial SSI, deep SSI, or organ space infection [22]. While peristomal skin infection is a widely reported surgical complication in this cohort [23-26], the present study indicates that the rate of periprosthetic infection and both deep and superficial SSI in colostomy patients undergoing TKA is no different compared to the control.

To our knowledge, this study marks the first attempt to address the influence of colostomy on postoperative outcomes following primary TKA. While this study presents novel findings, there is ample opportunity for further exploration into the influence of colostomy on TKA outcomes. Further study of mid- and long-term outcomes in this population would help to better direct their surgical management. While we found no difference in periprosthetic fracture or dislocation or implant mechanical failure, these outcomes are better studied beyond the initial hospital stay.

This study has limitations that must be acknowledged. The NIS database includes only inpatient data, so long-term complications of TKA in colostomy patients were not feasible within this study. Relying on ICD-10 codes from the NIS database for data retrieval poses a risk of coding errors that might undermine the accuracy and completeness of the gathered information. Also, due to data collection from multiple institutions, variations in blood transfusion threshold, surgical practices, and postoperative protocols are expected, potentially influencing the outcomes observed in this study. In addition, the NIS database does not afford access to laboratory results, like serum creatinine and hemoglobin levels which may provide meaningful information regarding the high incidence of blood loss anemia, blood transfusion requirement, and AKI in colostomy patients. Similarly, hospital protocols for blood transfusion could not be accessed and criteria likely vary at an institutional level. Despite these limitations, the NIS database’s large sample size enhances statistical power that allows for the identification of associations with heightened confidence. Additionally, the NIS database contains data from multiple institutions across 48 states, thereby enhancing its generalizability.

## Conclusions

Patients with colostomies who undergo primary TKA are at increased risk of experiencing AKI, blood loss anemia, and blood transfusion requirements during the immediate postoperative period compared to the general population. Despite the perceived risk of postoperative infection in colostomy patients, this patient population is not at an increased risk of developing periprosthetic infection, superficial SSI, or deep SSI following TKA. Future studies should address the impact of colostomy on the long-term outcomes following TKA.
